# Complexity of crack front geometry enhances toughness of brittle solids

**DOI:** 10.1038/s41567-024-02435-x

**Published:** 2024-03-22

**Authors:** Xinyue Wei, Chenzhuo Li, Cían McCarthy, John M. Kolinski

**Affiliations:** 1 Institute of Mechanical Engineering, School of Engineering, EPFL, Lausanne, Switzerland; 2 Williams College, Williamstown, MA USA

**Keywords:** Soft materials, Structural materials

## Abstract

Brittle solids typically fail by growth and propagation of a crack from a surface flaw. This process is modelled using linear elastic fracture mechanics, which parameterizes the toughness of a material by the critical stress intensity factor, or the prefactor of the singular stress field. This widely used theory applies for cracks that are planar, but cracks typically are not planar, and instead are geometrically complex, violating core tenets of linear elastic fracture mechanics. Here we characterize the crack tip kinematics of complex crack fronts in three dimensions using optical microscopy of several transparent, brittle materials, including hydrogels of four different chemistries and an elastomer. We find that the critical strain energy required to drive the crack is directly proportional to the geodesic length of the crack, which makes the sample effectively tougher. The connection between crack front geometry and toughness has repercussions for the theoretical modelling of three-dimensional cracks, from engineering testing of materials to ab-initio development of novel materials, and highlights an important gap in the current theory for three-dimensional cracks.

## Abstract (web summary)

Experiments probing three-dimensional crack propagation show that the critical strain energy needed to drive a crack is directly proportional to its geodesic length. This insight is a step towards a fully three-dimensional theory of crack propagation.

## Main

The propagation of a crack in a brittle solid often leads to material or structural failure—where the engineering utility of the object is completely lost—with important consequences for safety and cost. It is therefore essential that we understand, and ultimately aim to predict, when a crack might form and lead to fracture. Currently, linear elastic fracture mechanics (LEFM)^1–3^ is used throughout engineering science to model cracks; however, LEFM is based on the assumption that a crack is predominantly planar. Furthermore, energy-based analysis such as that used by Griffith^2,4,5^ can be carried out even for non-planar cracks, albeit only when the geometry is known. Planar cracks are at odds with observations and, indeed, our everyday experience—when one snaps a bar of chocolate in two or accidentally drops a glass, the fracture surface often appears corrugated or textured, and non-planar. Indeed, most crack surfaces are punctuated with localized features^6^ such as lances or ridges^7–11^, mist and hackle^12^, microbranches^13–15^ and crack nets^16,17^, depending on the stage of crack growth. Such pronounced deviations from a planar crack surface may be a consequence of instability under mixed-mode loading conditions^7,18–24^. These surface features are not perturbations to a flat crack, but instead they fundamentally change the crack front geometry, and are thus incompatible with existing three-dimensional (3D) perturbation methods^25–28^. Indeed, while path selection for planar cracks is generally determined by two ad hoc criteria^29–33^, in three dimensions there are no universally accepted path selection criteria, despite the tendency of cracks in polycrystalline solids to propagate between adjacent crystal grains^34^. Recent experiments show that step-like features can enhance the amount of energy dissipated during crack propagation^35–37^, but in situ 3D data for generally complex crack tips are lacking. Indeed, how geometric features at a crack front might alter the toughness of brittle solids remains unknown due to a dearth of experimental data on 3D crack configurations.

Here we use optical imaging methods to measure the 3D crack tip kinematic data in several brittle materials with high precision near the crack tip. The data are collected in situ using confocal microscopy while the sample is under load. We monitor the crack tip opening displacement (CTOD) to characterize the stress field at the crack tip, and we directly measure the geodesic length of the crack front using the imaging data. By comparing the loading state as assessed from the CTOD data and the crack front geometry from the geodesic length of the crack, we find that there is a direct, linear relationship between these two quantities, which appears to be a universal character among the materials we test. Furthermore, we find that the constant of proportionality is less than expected for pure energy dissipation along the crack front, suggesting that a diffuse damage region accompanies the highly localized dissipation occurring along the crack front, consistent with recent measurements of a fracture process zone in this material^38^. Finally, we identify localized crack advance along the crack front before global crack propagation, demonstrating that the crack can be locally critically loaded, and fractionally advance in three dimensions out of the crack plane, without globally failing.

Direct imaging with a confocal microscope is used to record the crack tip kinematics in brittle polyacrylamide hydrogels of various gel chemistries, as well as an elastomer (polydimethylsiloxane (PDMS)). To obtain contrast, the material sample is dyed with fluorescent dye, as described in detail in Methods. An edge crack is cut near the centre of the sample, extending approximately halfway across the sample width in the crack propagation direction. The initial incision generates crack tip complexity possibly due to the out-of-plane shear loading condition imposed during cutting. The sample and loading apparatus are then immersed in a water bath on the scanning stage of a confocal microscope. With the confocal microscope, only fluorescence emission from the focal plane is recorded on the detector. A resonant-galvo or galvo-galvo mirror pair is used to scan the beam and construct the planar image via rastering.

**Fig. 1:**
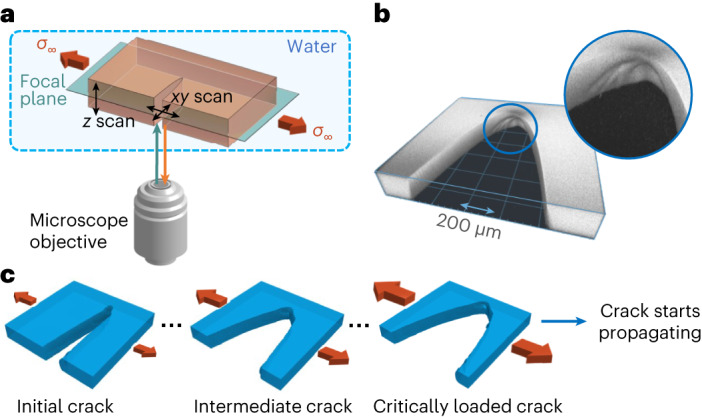
Loading and imaging complex 3D cracks. **a**, Schematic of the experimental setup. Confocal microscopy is used to image fluorescent light emitted from the sample in the focal plane. The experiment is carried out in water to match the optical index. Remote mode-I tensile loading is applied. **b**, A volumetric reconstruction of a complex crack tip near the critical stress required for propagation. The geometry of the crack tip is clearly not symmetric through the thickness, as can readily be seen by the features on the crack surface. **c**, Volumetric reconstructions of the same crack at different values of the applied strain demonstrate the experimental procedure: a complex crack tip is loaded with incrementally more strain, and allowed to equilibrate. Strain steps are kept very small to ensure resolution of the value of strain immediately below that for crack propagation, defining the critically loaded crack geometry.

The sample is initially unloaded. A small strain increment (typically less than one percent) is applied and a scanned image stack is recorded. Subsequently, a further strain increment is applied and a scanned image stack is recorded. This process of incrementing the strain and recording a scanned image stack is repeated until the crack propagates outside of the field of view, typically during loading. The propagating crack ruptures through the entire sample, leading to its global failure. The imaging setup is shown schematically in Fig. 1a. The volumetric reconstruction of a typical complex crack tip is shown in Fig. 1b. The loading sequence is depicted schematically with reconstructions of the sample volume around a loaded crack in Fig. 1c.

**Fig. 2:**
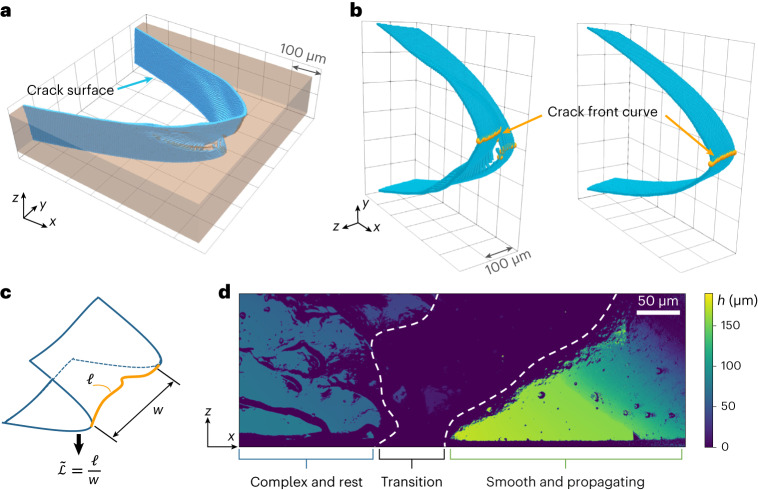
Complex and simple cracks. **a**, A 3D reconstruction of a crack with the crack surface highlighted in blue. **b**, A complex crack surface (left) is compared with a simple crack surface (right). The complex surface is the same crack as in **a**. **c**, Graphical definition of $\tilde{{{{\mathcal{L}}}}}$. The normalized crack front length is defined as $\tilde{{{{\mathcal{L}}}}}=\frac{\ell }{w}$, where *ℓ* is the geodesic crack front length and *w* is the measured sample thickness. **d**, A posteriori profilometry image shows the complex crack, loaded statically and at equilibrium until the critical strain is applied. The crack transitions to a smooth and propagating crack geometry once it reaches a critical loading value. Source data

During the loading process, the crack typically remains globally stationary. A complex crack tip and a smooth crack tip are shown, with only the fracture surface of the loaded sample depicted, in Fig. 2a. To parameterize the complexity of the crack tip, the geodesic length of the crack *ℓ* is normalized by the sample thickness *w* to form the normalized crack front length $\tilde{{{{\mathcal{L}}}}}$. Assessment of the error in the measurement of $\tilde{{{{\mathcal{L}}}}}$ arising from the finite resolution of the confocal microscope and segmentation of the images is described in Methods. These quantities are depicted schematically in Fig. 2b. After the crack has begun to propagate, it typically does so as a smooth, coherent crack, for which $\tilde{{{{\mathcal{L}}}}}\approx 1$. This process is not instantaneous, however; it instead occurs over some distance along the crack’s predominant propagation direction *x* as defined by the loading conditions. Initially, the crack surface has several complex features on it; it then undergoes an even more obscure crack advance in a transition region, before the crack front becomes smooth and nearly flat. This process is shown on a fracture surface for a typical complex crack in Fig. 2c, wherein the propagation stages are indicated by the brackets at the bottom of the profilometry image.

**Fig. 3:**
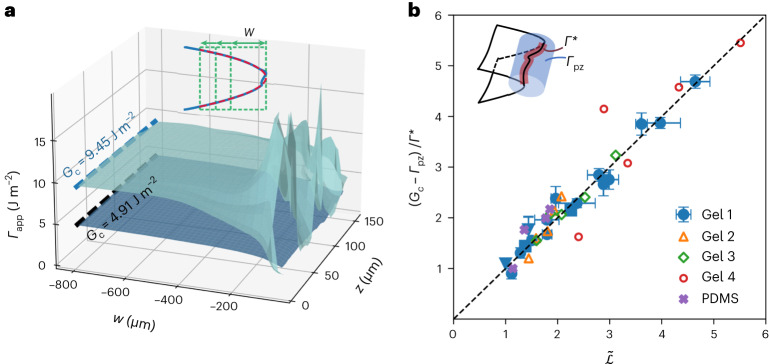
Apparent fracture energy and the crack tip opening displacement as a function of crack tip complexity. **a**, CTOD data are evaluated for each *z* slice by varying the evaluation window ${{{\mathcal{W}}}}$ (inset) and fitting a parabola to the CTOD. Apparent fracture energies *Γ*_app_ are then graphed as surfaces as a function of *z* and ${{{\mathcal{W}}}}$ for a simple (below) and complex (above) surface. In both cases, *Γ*_app_ converges to the background value *G*_c_. **b**, For various materials, the normalized critical strain energy release rate (*G*_c_ − *Γ*_pz_)/Γ^*^ increases in proportion with the crack tip complexity, measured by the normalized geodesic crack length $\tilde{{{{\mathcal{L}}}}}$. The square and the inverted triangle for gel 1 represent sample thicknesses of 100 μm and 380 μm, respectively. The linear proportionality is explained by the energy partition depicted schematically in the inset. The strain energy release rate is partitioned between bond scission at the crack tip, Γ^*^, which scales with $\tilde{{{{\mathcal{L}}}}}$, and a constant process zone fracture energy *Γ*_pz_. The evaluation of Γ^*^ and *Γ*_pz_ is described in Methods. Error bars are defined from *n* = 31 segmentations for $\tilde{{{{\mathcal{L}}}}}$. The sample number for *G*_c_ is provided in the data repository^48^. Better segmentation achieved with the embedded fluorophore in gels 2–4 and the PDMS generates error bars comparable to the symbol size for $\tilde{{{{\mathcal{L}}}}}$. Data are presented as mean values ± s.d. Due to the large CTOD, a tile acquisition was required for gels 2–4 and the PDMS sample; thus, no error bars are reported for these data. Source data

The CTOD is directly measured from the image stacks and volumetric reconstructions of the in situ crack opening profiles for each *z* slice. A parabola is fit to the CTOD as a function of both *z* and the window size, ${{{\mathcal{W}}}}$, yielding a prefactor *a* such that *x* = −*ay*^2^. From linear elastic fracture mechanics, the prefactor *a* is related to the mode-I stress intensity factor inversely as ${K}_{\rm {I}}=\sqrt{9\pi /(8a)}\mu$, where *μ* is the shear modulus, assuming incompressible material. The energy release rate $G={K}_{\rm {I}}^{2}/(3\mu )$, and by energy balance, the apparent fracture energy is *Γ*_app_ = 3π*μ*/(8*a*). Whereas for a complex crack tip the apparent fracture energy varies substantially near the crack front, it is very smooth and consistent near the crack tip for a smooth crack, and in both cases converges on a unique background value *G*_c_, as shown for two crack fronts with different complexity in Fig. 3a. When the critical stress intensity factor *G*_c_ is plotted as a function of the normalized geodesic crack length $\tilde{{{{\mathcal{L}}}}}$, it is observed to increase linearly from a fixed value of 4.9 J m^−^^2^ for gel 1, which is the baseline fracture energy of the hydrogel.

The observed linear increase is not simply given by the product of the crack front length and the baseline fracture energy, however; indeed, this product vastly over-predicts the observed *G*_c_ values. To account for the observed ${G}_{{{{\rm{c}}}}}(\tilde{{{{\mathcal{L}}}}})$ trend, the total fracture energy must be partitioned according to a distributed process zone, *Γ*_pz_, and the fracture energy of the bond rupture at the crack tip, Γ^*^. The scale of the process zone, the distance from the crack tip at which the clean CTOD deviates from the LEFM prediction^38^, is substantially larger than the variation of the crack tip position in the *x* − *y* plane, and thus it is assumed to be unaffected by the shape of the crack front curve, whereas the dissipation at the crack tip increases in proportion to the normalized crack front length, defined along the arclength, *s*. This scenario is depicted schematically in Fig. 3b, inset. The total fracture energy is thus given by

1 $${\it {\varGamma}} ={G}_{{{{\rm{c}}}}}(\tilde{{{{\mathcal{L}}}}})={\it {\varGamma} }_{{{{\rm{pz}}}}}+\int\nolimits_{0}^{\tilde{{{{\mathcal{L}}}}}}{\rm {d}}s{\it {\varGamma} }^{* }={\it {\varGamma} }_{{{{\rm{pz}}}}}+\tilde{{{{\mathcal{L}}}}}\times {\it\varGamma }^{* }.$$

Prior measurements establish that *Γ*_pz_ = 3.13 ± 0.44 J m^−2^ and Γ^*^ = 1.77 ± 0.44 J m^−2^ for gel 1 (ref. ^38^). A linear fit to the data in Supplementary Fig. 1a,b yields *Γ*_pz_ = 3.81 J m^−2^ and Γ^*^ = 1.22 J m^−2^, as shown in the black line. While these values fall outside the range of the error bars as determined by linear extrapolation, the observation that *Γ*(*x*) drops faster than linear^38^ indicates a smaller Γ^*^ than the value calculated from linear extrapolation. It is important to note that while this phenomenological expression relates the normalized strain energy release to the geodesic crack length across several materials and a wide range of $\tilde{{{{\mathcal{L}}}}}$, we did not exhaustively evaluate all candidate geometric expressions.

The five materials tested here have different values of *Γ*_pz_. The values for Γ^*^ are the same for all gels, and nearly zero for the PDMS, as explained in Methods. To compare these materials in an aggregate graph, we must normalize *G*_c_ by Γ^*^ after subtracting *Γ*_pz_; in this way, we arrive at a non-dimensional measure of the energy release rate that we can compare with the non-dimensional geodesic crack length $\tilde{{{{\mathcal{L}}}}}$. All recorded data are normalized in this manner and plotted in the graph shown in Fig. 3b.

**Fig. 4:**
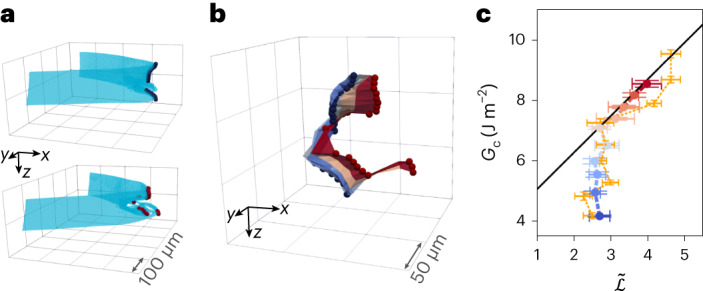
Subcritical crack front evolution. **a**, The crack front (marked by dots) is not static, but develops as the applied strain increases from the upper to the lower panel. **b**, The coloured curves trace the crack tip at incremental applied strains 1–6 corresponding to the colour code in **a** and **c**. Curves are registered as described in Methods. **c**, The applied strain energy during the loading process is plotted as a function of $\tilde{{{{\mathcal{L}}}}}$. Matching the colour code in panel **b**, there is little variation in $\tilde{{{{\mathcal{L}}}}}$ during the initial loading process. Until reaching the critical value *G*_c_, $\tilde{{{{\mathcal{L}}}}}$ increases to keep the globally critical state. A completely independent sample is shown in yellow points, which reaches the same trend. The critical strain energy boundary at which all cracks are observed to propagate seems to be a universal bound, as it is not exceeded during the loading process in the intermediate or the final applied strain increments. Error bars are defined from *n* = 31 segmentations for $\tilde{{{{\mathcal{L}}}}}$ and *n* = 50 samples of *G*_c_. Sample number for *G*_c_ is provided in the data repository^48^. Data are presented as mean values ± s.d. Source data

While the data shown in Fig. 3b represent cracks that propagated and led to global failure of the sample under test, the time series of crack volumes indicates that crack propagation can occur partially along the crack front, as shown for a representative crack front at two different strain increments in Fig. 4a. The critical loading state is not uniform along the crack front, but instead can be locally critical, but globally stable; such pinning has been observed for planar cracks propagating along a surface with heterogeneous fracture energy^39,40^ and is similar to the non-uniform crack advancement observed in the debonding of adhesives^41–43^. An instance of fractional crack front propagation is shown by the surface swept out by the crack front at incremental strain steps. Throughout the loading, the crack is locally pinned over a narrow range of *z*, but notably advances for other values of *z*, as shown in Fig. 4b.

During the loading process, the corresponding $\tilde{{{{\mathcal{L}}}}}$ data remain nearly constant as the subcritical strain energy density *G* increases with increasing strain, as shown for the loading sequence in Fig. 4c. Here, the coloured points correspond to the lines graphed in Fig. 4b. A completely separate sample, subjected to a similar loading increment, is shown in the yellow points in Fig. 4c. Strikingly, *G* converges on the value of *G*_c_ for the relevant $\tilde{{{{\mathcal{L}}}}}$ value at which the crack should be globally unstable, but by fractionally advancing, $\tilde{{{{\mathcal{L}}}}}$ can increase as necessary to keep the crack just below the globally critical state, until it destabilizes and the sample catastrophically fails.

**Fig. 5:**
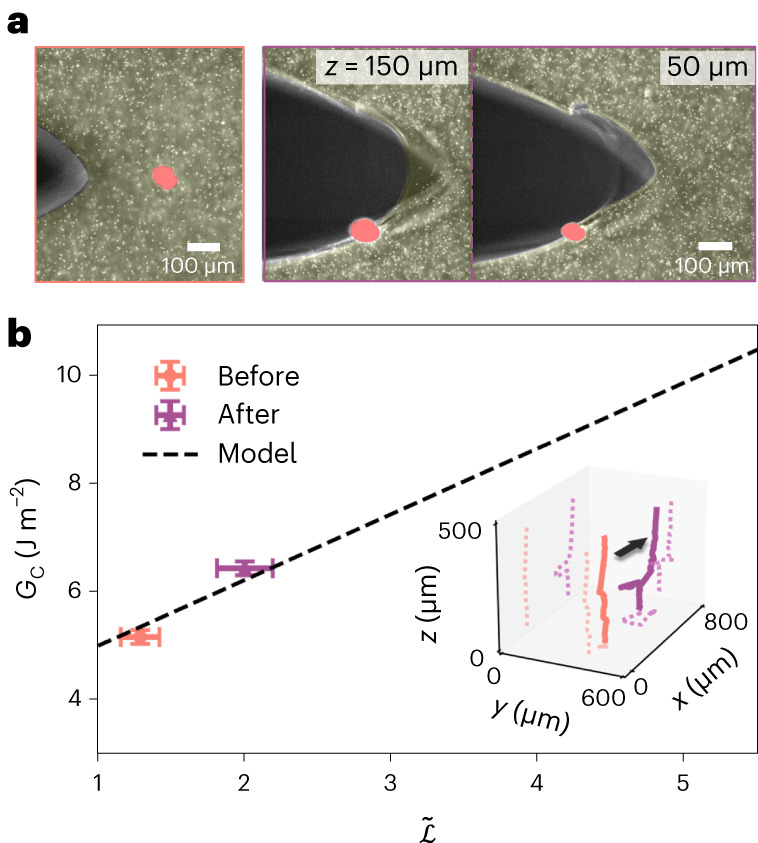
Rigid inclusion drives crack plane symmetry breaking in a 400 μm hydrogel. **a**, Left, a micrograph of a quasi static planar crack propagating towards an embedded rigid Nylon inclusion (polyamid, diameter 50 μm). The hydrogel and the inclusion are highlighted with light yellow and red. Right, two crack micrographs recorded at different values of *z* from the sample bottom after encountering the inclusion. The planar symmetry is broken, by a large material filament that joins two distinct crack planes near the centre of the sample. White points are sterically bound 1.1 micron latex particles. **b**, *G*_c_ versus $\tilde{{{{\mathcal{L}}}}}$ before and after the crack encounters the inclusion. Inset, 3D crack fronts extracted from the two volumetric images in **a**; the crack advances in the direction indicated by the arrow, corresponding to the remote tensile loading symmetry. $\tilde{{{{\mathcal{L}}}}}$ is measured from the crack front geometry and *G*_c_ is evaluated from parabolic fitting of the far-field CTOD (*x* < 180 μm, Supplementary Fig. 2). The error bars in $\tilde{{{{\mathcal{L}}}}}$ correspond to the standard deviation of five independent crack tip identifications, and the error bars in *G*_c_ are adapted from average standard deviation of confocal measurements presented in Fig. 3. Data are presented as mean values ± s.d. Source data

Thus far, we have shown that crack front complexity can increase sample toughness. To demonstrate the engineering consequences of the observed relationship between crack front complexity and toughness, we adopt a strategy to exploit this relationship with elastic heterogeneity, motivated by earlier work^27,33,44–46^. To this end, we embed a relatively rigid Nylon particle in the gel. The crack begins propagating slowly toward the particle such that the crack path is incident on the particle, as shown in the micrograph in Fig. 5a, left. Upon progressing past the particle, the crack is no longer planar, but instead planar symmetry is broken; this can readily be seen in the micrographs recorded in Fig. 5a, at right. Upon applying our analysis of the CTOD and normalized crack front length to measure *G*_c_ and $\tilde{{{{\mathcal{L}}}}}$, respectively, we find that the data before and after the encounter with the particle fall upon the same line as was observed for intrinsic crack tip complexity as shown in Fig. 5b. The particle doubles $\tilde{{{{\mathcal{L}}}}}$, generating a 25% increase in *G*_c_. Such planar symmetry breaking, triggered by elastic inclusions, likely occurs in composite solids^45,46^, with large numerical density of inclusions; indeed, stress intensity factors for crack fronts that encounter such inclusions are known to be larger^44^. This result may relate to the toughening of fibre composites, which can also generate toughness with other mechanisms, including fibre bridging^45^.

With direct 3D imaging data, we have shown that a complex crack front generates a tougher material overall, without altering the material’s fracture toughness, in a systematic way. We find that the toughness enhancement scales linearly with the geodesic crack length when dissipation in the process zone is accounted for. Purely by enhancing the geometric complexity of the crack tip curve, more strain energy is required for the crack to advance. Indeed, the complexity plays an even more subtle role—it can generate a condition where the crack is globally stable, but still advances along a portion of the crack front; thus, the local crack front can be unstable, whereas the global crack configuration remains stable, in a manner reminiscent of crack front pinning observed in planar systems with heterogeneous fracture energy^39,40^.

These measurements are consistent with two dimensional measurements of a complex crack front in the same material with a single facet^37^, but are now extended to arbitrary crack front complexity, and enable the first in situ crack front length measurement for a subcritical or critically loaded crack before propagation. As there is currently no theory for a crack that has dramatically broken planar symmetry, the essential phenomenology uncovered here cannot be explained by an existing theoretical framework—while *G*_c_ can be used to characterize toughness enhancement for mechanisms ranging from rigid inclusions^44^ to fibre bridging^46^, a priori, it offers no predictive power of how much an increase in geodesic crack length will enhance *G*_c_. Indeed, the fundamental reason that the crack front length toughens the brittle solid remains unknown, as does the extent to which one should expect the toughening to saturate. Whereas here we have identified a crack that is nearly twice as tough as a smooth crack in the material we are testing, there is no reason that we should not anticipate an even greater enhancement in toughening purely by geometry.

The fractional advance of the crack front is all the more puzzling, as the critical fracture condition according to LEFM is not local, but instead related to the singular stress field that develops in the approach to the crack tip. Our measurements suggest that instead, the critical crack growth condition can be local once the planar symmetry of the crack is broken. This begs the question: what is the physical condition governing 3D crack growth? It may be that local energy balance in *z* is satisfied along the crack front, as suggested in prior work^47^; however, this is a planar theory for crack advancement and may require nontrivial extension to fully capture the 3D elastic fields near a non-planar crack.

The measurements we have carried out suggest that purely by enhancing the geometric features at the tip of a crack, a material can be tougher—superficially, this picture is at odds with the Griffith picture, which suggests that the fracture energy is a material property, whereas here, if one does not carefully account for the crack tip geometry, the fracture energy appears to take on different values. The consequences of our measurements are widespread—for example, can we manipulate a crack front to make it more complex, and is this done already in certain composite materials? Earlier work suggests a means to address this question using analysis built upon LEFM^44^. Materials testing can also be informed by our measurements. Engineers have long known that the initial state of the crack must be carefully controlled for a reliable measurement of the critical stress intensity factor, *K*_Ic_; this is typically accomplished in the case of compact tension specimens by fatigue loading of the crack until it is planar; however, our measurements highlight the importance of care in carrying out materials testing, as any geometric deviation from a planar crack front may lead to a mismeasurement, and dangerous over-estimation, of material toughness. Our observations that *G*_c_ is proportional to $\tilde{{{{\mathcal{L}}}}}$, that this curve serves as an attractor for cracks, and that rigid inclusions can manipulate the location of a crack in the ${G}_{{{{\rm{c}}}}}\!\!-\!\!\tilde{{{{\mathcal{L}}}}}$ space provide insight into the physics of fracture and tools for the engineering scientist to realize material toughness.

Using the brittle hydrogels and the PDMS elastomer as proxy materials, we have investigated the propagation of cracks that break planar symmetry, and push us away from the existing LEFM picture of planar fracture. Such symmetry breaking leads to a geometric toughening effect that can be accounted for by correctly summing contributions to fracture energy in the materials. Such behaviour is expected to be universal if the process zone and crack tip fracture energies are known for any material, and thus our observations are anticipated to be universal for brittle or even somewhat ductile materials. Depending on the geometry of the crack front, fractional advancement of the crack can occur, generating a challenge for existing theoretical approaches to describe the mechanics of 3D cracks. Nevertheless, the critical strain energy density required to drive a crack appears to be bounded, based on the fractional advance of cracks along their length. What sets the limit of how much toughening can be achieved by geometry remains to be established, as does the persistence of toughening under sustained load.

## Methods

### Sample preparation and material characterization

Gel samples are polymerized from monomer solution in ultra-pure water consisting of 13.8% (weight per volume) acrylamide monomer, of which the bis-acrylamide cross-linker concentrations are 2.67%, 1.87%, 0.81% and 0.35% for gels 1 to 4, respectively. A 0.2% solution of ammonium per sulfate (APS) is used to initiate polymerization at a ratio of 1.8% by volume, and Tetramethylethylenediamine (TEMED) catalyses polymerization at 0.18% by volume. Two fluorescent dyes, Rhodamine 6G and Fluorescein o-acrylate, are used for contrast during imaging. Neither dye alters the mechanical properties of the gels. The data points for gel 1 in Fig. 3b are obtained using samples with Rhodamine 6G dye, while the data for the rest of the gels are obtained using samples with Fluorescein o-acrylate dye. Fluorescein o-acrylate dye is added to the monomer solution before polymerization, while Rhodamine 6G is applied after. All chemistry is obtained from Sigma Aldrich.

The monomer solution (with 1% of 1 mg ml^−1^ Fluorescein o-acrylate dye if used) is degassed for 10 min in vacuum before introduction of the APS and TEMED. Following degassing, the APS and TEMED are added, and the solution is gently mixed before being poured onto a glass plate. Plastic spacers are used to obtain different sample thicknesses, which are 190 μm for all the gel chemistries, with additional 100 μm and 380 μm for gel 1. Spacers are placed at the four corners of the glass plate, and a second plate is carefully rested atop the solution and the spacers. The edges of the sample are sealed with plastic cling film, and a weight is placed over the spacers to ensure a consistent sample thickness. The polymerization reaction is allowed to run for at least four hours before the material is handled.

After polymerization is complete, 3 × 1 cm rectangular samples are cut from the hydrogel using a razor blade. Care is taken to ensure that the sample edges are free of notches or geometric defects. To dye the gels samples with Rhodamine 6G, we place the samples in a large bath of 2 × 10^−4^ mol l^−1^ solution of the dye and allow the solvent to reach equilibrium.

Polydimethylsiloxane (PDMS) Sylgard 184 silicone elastomer are obtained from Sigma Aldrich. Samples are prepared with the 10:1 ratio of the prepolymer base and the curing agent, fluorescently dyed with 0.1% TP-3400, mixed and degassed at room temperature, and cured at 80 ^°^C for 4 h.

A uniaxial tensile test is performed to characterize the material properties. The engineering stress is evaluated with respect to the stretch as shown in Supplementary Fig. 3. The loading curves for the gel materials are fitted to the neo-Hookean material model^49^, which gives the shear modulus of 37.53 ± 0.01 kPa, 13.07 ± 0.009 kPa, 5.55 ± 0.005 kPa and 3.12 ± 0.002 kPa for gels 1 to 4. The loading curve of PDMS is fitted to the generalized neo-Hookean material model due to strain stiffening. The fitting gives the shear modulus of PDMS of 0.323 ± 0.001 MPa.

### Evaluation of *G*_c_– ℒ^~^

In this work, we find that *G*_c_ increases proportionally with the crack tip complexity $\tilde{{{{\mathcal{L}}}}}$, where we observe no thickness-dependency, as shown in Supplementary Fig. 2a. This proportionality is explained by the energy partition between the process zone dissipation *Γ*_pz_ around the crack tip and the bond scission energy Γ^*^ at the crack tip, and *G*_c_ is normalized as (*G*_c_ − *Γ*_pz_)/Γ^*^, as shown in Fig. 3b. The non-normalized ${G}_{{{{\rm{c}}}}}-\tilde{{{{\mathcal{L}}}}}$ data is plotted in Supplementary Fig. 1b,c. *Γ*_pz_ is evaluated as the intersect from the linear fittings to the ${G}_{{{{\rm{c}}}}}-\tilde{{{{\mathcal{L}}}}}$ data of each gel, which are 3.81 J m^−2^, 2.21 J m^−2^, 1.41 J m^−2^, 0.49 J m^−2^, for gels 1 to 4, respectively. The slope of the linear fitting to gel 1 gives Γ^*^ = 1.22 J m^−2^. The energy related to bond breaking, Γ^*^, is taken to be the same for the different gel chemistries, due to the same monomer concentrations. Thus, we use the Γ^*^ value from gel 1 for the rest of the gels, as gel 1 has the most data points. For PDMS, due to the small fractal cohesive length, *Γ*_pz_ ≈ 0 and Γ^*^ is 57.48 J m^−2^, which is the *G*_c_ of a clean crack (the first data point Supplementary Fig. 2c, the closest point to a clean crack).

### Confocal microscopy and mechanical load application

To capture the in situ 3D crack data, an inverted Leica DMi8 Confocal microscope is used with a HC PL Fluotar ×10 air immersion, or HC Fluotar ×25 water immersion, objective. The excitation laser wavelength and the bandwidth of the hybrid detector are set to match the excitation and emission spectrum of the fluorescent dyes. An edge notch is cut halfway across the sample breadth using a Victorinox Swiss knife (Classic SD) near the centre of the sample’s long edge. The sample is then mounted into the sample grips of a custom loading apparatus and immersed in a water bath atop the microscope stage. The loading apparatus drives the grips symmetrically with a servo motor, thus ensuring that the crack remains in the microscope field.

### Profilometry and height profiles

Profilometry measurements are performed after the gel samples are fully broken. To obtain good optical reflection and avoid the dehydration problem, the fracture surfaces are cast onto Polyvinyl siloxane (PVS; Zhermack), and the height profiles are taken using Nikon TI eclipse microscope and Nikon 10× Mirau objective, with a step size of 50 nm. Profilometry height profiles are analysed to evaluate the surface roughness on a sustantially smaller scale than the confocal microscope, and thus provide a bound on surface roughness. In the smooth region in the main text in Fig. 2c, the height profile of three random cuts are shown in Supplementary Fig. 4b,c. The height profiles are separated into the high and low-frequency parts in Supplementary Fig. 4c, using a low-pass linear filter with the cutoff frequency of 0.17 radians per second, where 0.17 = 1/(2 × 4.28) and 4.28 μm is the *z* sectioning of the applied objective on the confocal microscope. The h-high corresponds to the measured crack front length from confocal microscopy, while the h-low contributes to the length from the missing roughness features. The low-frequency part is smaller than the high frequency parts, indicating that the confocal step size captures the key trend of the crack front curves.

### Image processing

To obtain the fully segmented image data, three software packages were used: Imaris, Fiji^50^ and scikit-image^51^. The image processing workflow is shown in Supplementary Fig. 5. A 3D Gaussian filter was used to remove salt and pepper noise, and thresholding operations were carried out to obtain a first segmentation; this was followed by morphological operations to obtain a completely segmented 3D volume. To control for processing-dependent variability of the resulting segmented image, the 3D Gaussian kernel size was varied from one to three pixels in the image plane, and two to four steps through the image stack. A total of five intensity levels were used to threshold the filtered image stacks, generating a total of 30 different processed stacks. Note that we use the Imaris software to segment the data manually with the same procedure, generating a total of 31 unique data pairs for each image stack. A final morphological processing step was applied to fill holes in the sample volume. From each of the stacks, a *G*_c_ − $\tilde{{{{\mathcal{L}}}}}$ data pair is generated, as shown in Supplementary Fig. 6a.

### Rigid inclusion

For the gel samples with embedded rigid Nylon inclusions, the concentration of the Nylon particles (diameter 50 μm; Dantec dynamics) is 0.05 wt%. At this concentration, only one inclusion remains within the field of view on average. The low seeding density ensures that the Nylon particles do not alter the mechanical response of the hydrogel material. The parabolic fit to the CTOD data before and after the crack encounters the rigid inclusion is shown in Supplementary Fig. 2. To minimize the effect of the rigid inclusion on the fittings, the parabolas are fitted to the CTOD which is away from the inclusion.

## Online content

Any methods, additional references, Nature Portfolio reporting summaries, source data, extended data, supplementary information, acknowledgements, peer review information; details of author contributions and competing interests; and statements of data and code availability are available at 10.1038/s41567-024-02435-x.

### Supplementary information

Supplementary Information Supplementary Figs. 1–6 and video description.

Supplementary Video A *z*-scan through the particle is shown, along with the segmented gel sample, to highlight the emergent complexity at the crack tip due to the crack–particle interaction.

### Source data

Source Data Fig. 2 Grid data for profilometry measurement in **c**.

Source Data Fig. 3 Surfaces in panel **a**, source data for mean and error of all points in **b**.

Source Data Fig. 4 Local propagation of the crack under increasing strain in panels **a** and **b**; source data for mean and error of all points in **c**.

Source Data Fig. 5 Source data and error for data in **b**.

## Data Availability

Figures are available via figshare. Segmented image data for all crack tips analysed in the manuscript can be found in the associated digital data repository^48^. Source data are provided with this paper.
