# Supplementary material for: Complexity of crack front geometry enhances toughness of brittle solids
Source: Nat Phys. 2024 Mar 22;20(6):1009–14. doi: 10.1038/s41567-024-02435-x (PMC11178495; doi:10.1038/s41567-024-02435-x)
Supplement: Supplementary file 1 — Supplementary Figs. 1–6 and video description. [file 41567_2024_2435_MOESM1_ESM.pdf]

nature physics

Article https://doi.org/10.1038/s41567-024-02435-x

Complexity of crack front geometry enhances toughness of brittle solids

In the format provided by the authors and unedited

Supplementary materials

Figs. S1 to S6
Video 1

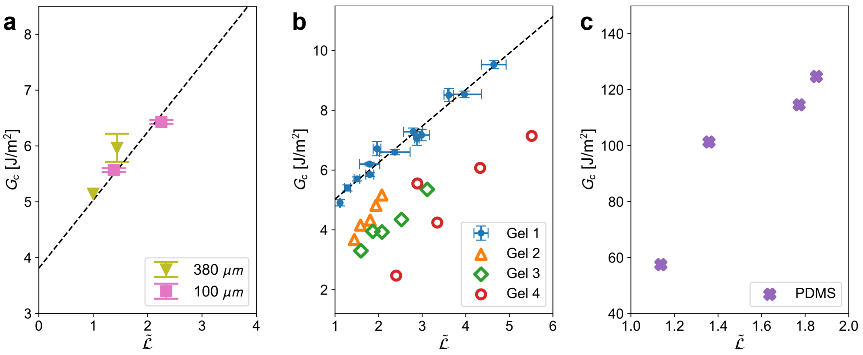

Fig. S1 **a**, $G_c - \tilde{\mathcal{L}}$ plot of Gel 1 with various sample thicknesses. The black dashed line is the linear fitting of the Gel 1 data. **b**, $G_c - \tilde{\mathcal{L}}$ plot of different gel chemistries. The black dashed line is the same as in **a**. **c**, $G_c - \tilde{\mathcal{L}}$ plot of PDMS. Error bars are defined from $n = 30$ segmentations for $\tilde{\mathcal{L}}$, and $n = 50$ samples of G_c. Better segmentation achieved with the embedded fluorophor in gels 2-4 and the PDMS generate error bars comparable to symbol size for $\tilde{L}$. Data are presented as mean values $\pm$ standard deviation. Due to the large CTOD, a tile acquisition was required for gels 2-4 and the PDMS sample; thus, no error bars are reported for these data.

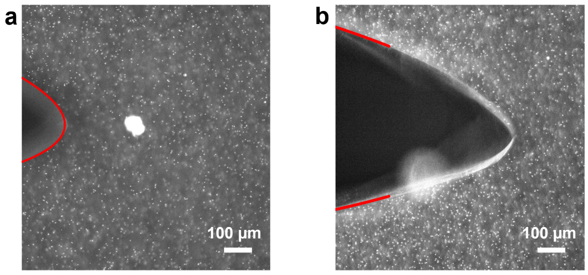

Fig. S2 **Fitted data for CTOD measurement of crack interacting with rigid particle.** Fitted parabola of cracks **a** before and **b** after encountering the rigid inclusion, overlaid on the image slices. Only the most left CTOD are used in the fitting in order to minimize the influence of the rigid inclusion.

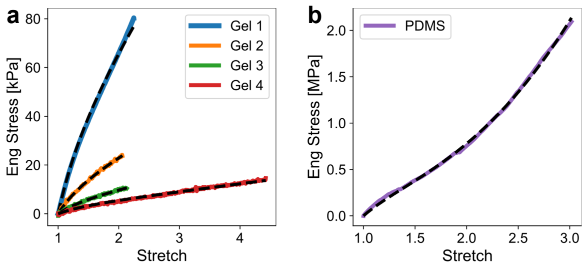

Fig. S3 Uniaxial tensile engineering stress-stretch curves. **a**, Hydrogels with different chemistries. The loading curves are fitted to the neo-Hookean material model (dashed curves). **b**, PDMS. The loading curve is fitted to the generalized neo-Hookean material model[1] (dashed curve).

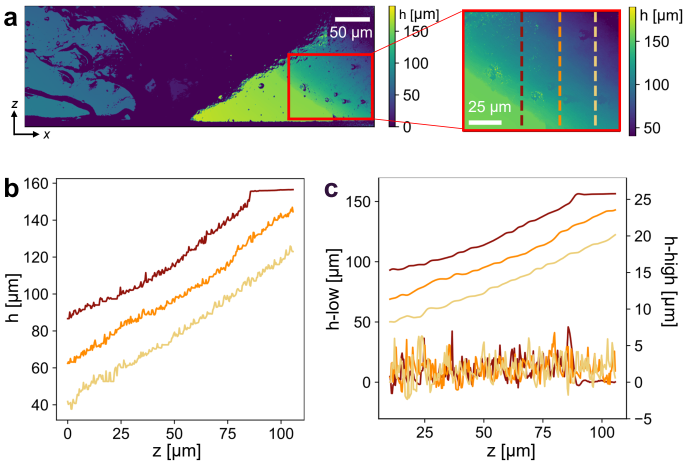

Fig. S4 Height profile of a typical fracture surface. **a**, Profilometry measurement of the fracture surface with the zoom-in view on the right. The highlighted dashed lines in the right panel are where the height profiles are sampled. **b**, Height profiles of he dashed lines in b) with corresponding colors. **c**, The height profiles is separated into low and high frequency part. The low frequency signal is obtained using linear low pass filter with a cutoff frequency of $1/(2 \times 4.28)$ radians/sec, where 4.28 μm is the z step size for most of the image stacks taken with confocal microscopy.

Supplementary Video

After the crack passes by a rigid inclusion, the crack front can become complex. In this video, we scan through the thickness of a sample where the crack front was made more complex after interacting with the particle. A filament of material connects the crack faces, enhancing toughness, and generating a complex crack tip geometry.

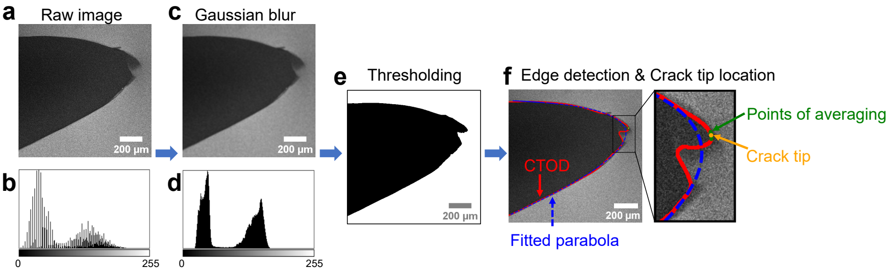

Fig. S5 Image processing and crack tip locating. a-b, A typical raw image of a crack and its intensity histogram. **c-d**, The image after Gaussian blur and its intensity histogram. **e**, The binarized image after thresholding. **f**, Edge detection of **e** gives the CTOD (red curve) plotted on the raw image. A parabola (blue dashed curve) is fitted to the CTOD. The zoomed-in view shows the crack tip location. The symmetric axis of the parabola gives the propagation direction. The crack tip (orange dot) is found by averaging a set of maximum points (green dots) within 3 µm along the propagation direction.

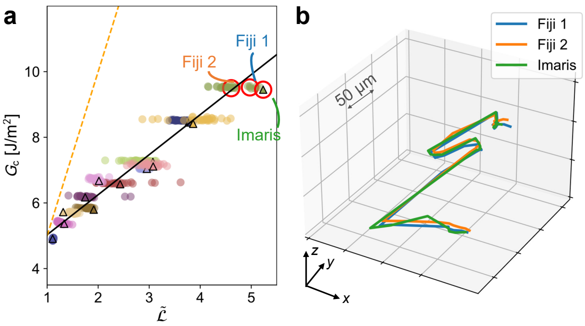

Fig. S6 Comparison of image processing methods. a, G_c **vs** $\tilde{\mathcal{L}}$ **for different segmentation method.** The same color represent the same image stack. The images are processed using either Imaris and Fiji softwares with different parameters for the 3D Gaussian blur and the fractional intensity levels used to threshold the image stacks, as described in the Materials and Methods. Segmentation carried out with Imaris is indicated by triangle symbols. Combinatorial variation of the Gaussian blur and threshold parameters is carried out with Fiji over a total of 31 unique parameter sets each image stack. Segmented volumes are compared with the raw images to ensure correspondence between the post-processes images and the original data. After segmentation, morphological operations are used to fill the holes in the sample and obtain the final segmented volume with the python package scikit-image[2]. **b**, Comparison of the same crack front curves segmented by three different methods as highlighted in **a**.

References

[1] Geubelle, P.H., Knauss, W.G.: Finite strains at the tip of a crack in a sheet of hyperelastic material: I. homogeneous case. Journal of Elasticity **35**(1-3), 61–98 (1994)

[2] Walt, S., Schönberger, J.L., Nunez-Iglesias, J., Boulogne, F., Warner, J.D., Yager, N., Gouillart, E., Yu, T.: scikit-image: image processing in python. PeerJ **2**, 453 (2014)
